# JMJD2B-induced amino acid alterations enhance the survival of colorectal cancer cells under glucose-deprivation via autophagy

**DOI:** 10.7150/thno.38087

**Published:** 2020-04-27

**Authors:** Juan Tan, Hao-Lian Wang, Jie Yang, Qian-Qian Liu, Chun-Min Li, Yun-Qian Wang, Lin-Na Fu, Qin-Yan Gao, Ying-Xuan Chen, Jing-Yuan Fang

**Affiliations:** 1 Division of Gastroenterology and Hepatology, Key Laboratory of Gastroenterology and Hepatology, Ministry of Health, State Key Laboratory for Oncogenes and Related Genes, Renji Hospital, School of Medicine, Shanghai Jiao Tong University; Shanghai Institute of Digestive Disease;145 Middle Shandong Road, Shanghai 200001, China.; 2 Department of Biochemistry and Molecular Cell Biology, Key Laboratory of Education Ministry for Cell Differentiation and Apoptosis, Institute of Medical Sciences, Shanghai Jiao Tong University School of Medicine. 280 South Chongqing Rd, Shanghai 200025, China.

**Keywords:** JMJD2B, CRC, amino acids metabolism, autophagy, LC3B

## Abstract

**Rationale**: Post-translational modifications have emerged as vital players in alterations to tumor metabolism, including amino acid metabolic reprogramming. Jumonji domain-containing protein 2B (JMJD2B) enhances colorectal cancer (CRC) cell survival upon glucose deficiency. In the present study, we hypothesized that JMJD2B affects tumor cell amino acid metabolism in CRC and consequently promotes survival of CRC cells upon glucose deprivation.

**Methods**: Non-target metabolic profiling was used to evaluate the roles of JMJD2B in CRC cell metabolism under glucose starvation. The roles of amino acid alterations induced by JMJD2B on CRC cell survival were determined by cell viability, immunoblotting, and clonogenic assays, and flow cytometry. The underlying mechanisms by which JMJD2B affected CRC cell metabolism were assessed using immunofluorescence staining, chromatin immunoprecipitation assays, electron microscopy in CRC cell lines, and using xenograft models. The correlation between JMJD2B and LC3B expression in human CRC specimens was assessed using immunohistochemistry.

**Results**: Profound metabolic reprogramming was detected in *JMJD2B* knockdown CRC cells under glucose deficiency, especially those involving amino acid metabolites. Silencing of *JMJD2B* reduced the levels of certain amino acids that were induced by glucose deficiency. Among these amino acids, asparagine (Asn), phenylalanine (Phe), and histidine (His) promoted CRC cell survival under glucose starvation when *JMJD2B* was knocked down. Mechanistically, downregulation of *JMJD2B* inhibited autophagy in CRC cells through epigenetic regulation of microtubule associated protein 1 light chain 3 beta (LC3B), and subsequently decreased intracellular amino acid (Asn, Phe, His) levels under glucose deprivation, thus suppressing the survival of CRC cells. Using a nude mouse xenograft model, we verified that inhibiting JMJD2B could decrease the levels of amino acids (Asn, Phe, His). In addition, the inhibitory effects of *JMJD2B*-knockdown on tumor growth and amino acids level were rescued by overexpression of *LC3B*. Furthermore, we observed that the high expression of LC3B was more likely detected in tissuses with high expression of JMJD2B (*P* < 0.001) in 60 human CRC tissues.

**Conclusion**: These results indicated that JMJD2B sustained the intracellular amino acids derived from autophagy in CRC cells upon glucose deficiency, partly through epigenetic regulation of *LC3B*, thus driving the malignancy of CRC.

## Introduction

Colorectal cancer (CRC) is a frequently diagnosed malignancy and is the second leading cause of cancer death, resulting in a significant economic burden, especially in the developing world 1. Therefore, it is important to better understand the molecular mechanisms that contribute to the aggressiveness of CRC. Although previous studies have identified multiple cellular processes that promote CRC tumorigenesis, including genomic and epigenomic changes, the precise mechanism remains unknown.

Jumonji domain-containing protein 2B (JMJD2B), also known as KDM4B (lysine demethylase 4B), mainly removes tridimethylation (me3/2) at the nine lysine (K) residues of histone H3 2. Our previous study showed that JMJD2B is highly expressed in CRC cancer tissues 3, and is overexpressed in a variety of tumors 2. Silencing of *JMJD2B* caused cell cycle arrest, apoptosis, and senescence of CRC cells, thus inhibiting their survival 3, 4. The abnormal growth of functional blood vessels associated with rapid cancer cell proliferation in solid tumors results in some regions within the tumors being temporarily or continuously under stress in an unfavorable microenvironment, particularly nutritional deficiency or hypoxia 5-7. The expression of JMJD2B was upregulated under glucose deficiency or hypoxia, and JMJD2B could promote the survival of CRC cells under these conditions 4, 8. However, it is unclear how JMJD2B promotes the survival of CRC cells under stress in the unfavorable tumor microenvironment.

Tumor cells can adapt to changes in their unfavorable microenvironment by increasing the utilization of amino acids. Amino acids are used as intermediate metabolites to synthesize important biological molecules, e.g., nucleotides, lipids, glutathione, and carbon units; they can also be oxidized in the tricarboxylic acid cycle (TCA) instead of glucose to produce more ATP and NADH; some could promote accumulating reductive glutathione (GSH) and reduce reactive oxygen species 9, 10. For instance, the serine biosynthesis pathway was activated under glucose deprivation conditions 11. Our previous study found that JMJD2B regulated many cellular processes and signaling pathways under hypoxia, in which cellular metabolic processes and metabolic pathways were the most significant part, including amino acid metabolism 3. Therefore, we hypothesized that JMJD2B might affect tumor cell amino acid metabolism in CRC and consequently promote cellular survival in CRC cells upon glucose deprivation.

In the present study, we detected marked metabolic reprogramming after *JMJD2B* knockdown under glucose deficiency conditions in CRC cells, with amino acid metabolites being the most affected by lack of JMJD2B. Metabolomic analysis showed that 27 amino acid-related metabolites were upregulated under glucose deprivation, of which 15 were downregulated by *JMJD2B* knockdown, including five amino acids. Among these five amino acids, asparagine (Asn), phenylalanine (Phe), and histidine (His) promoted CRC cell survival under glucose deprivation in a background of *JMJD2B* knockdown. Mechanistically, JMJD2B promoted autophagy during glucose deprivation to sustain intracellular amino acid levels (Asn, Phe, His) in CRC cells, via epigenetic regulation of microtubule associated protein 1 light chain 3 beta (LC3B). Collectively, our findings describe a new regulatory mechanism of glucose deprivation-mediated CRC metabolism, identifying JMJD2B as a promising target for CRC therapy.

## Methods

### Cell lines, plasmids, adenovirus, and lentivirus

Human CRC cell lines HCT116 and SW480 were purchased from the ATCC (the American Type Culture Collection, Manassas, VA, USA). All cell lines were grown in a humidified 5% CO_2_-containing atmosphere incubator at 37 °C. For glucose deficiency, 48 h after seeding, the cells were washed briefly using phosphate-buffered saline (PBS) and cultured in glucose-free Roswell Park Memorial Institute (RPMI) 1640 medium (Gibco BRL, Gaithersburg, MD, USA) for the indicated times. RPMI 1640 media without amino acids and glucose was purchased from US Biological (catalog no. #R9010-01, Swampscott, MA, USA). The amino acids were added into the amino acids-free and glucose-free medium for the indicated times as follows: Asn (2 mM, catalog no. #A4159), Phe (2 mM, catalog no. #P5482), His (2 mM, catalog no. #H5659), and hydroxy-proline (Hyp, 2 mM, catalog no. #H5534) 12; all of which were purchased from Merck (St. Louis, MO, USA). The siRNA-resistant JMJD2B wild-type plasmid (pCMV-HA-JMJD2B-WT), the H189A/E191Q mutant plasmid (pCMV-HA-JMJD2B-MT), and the pCMV-GFP-LC3B plasmid were purchased from GENEray Biotech (Shanghai, China). Short hairpin RNA (shRNA) adenovirus constructs targeting *JMJD2B* were purchased from OBiO Technology (Shanghai, China). The lentivirus targeting the *LC3B* gene was purchased from Genechem (Shanghai, China), which was used to establish HCT116 cells stably overexpressing LC3B.

### RNA interference

Small interfering RNAs (siRNAs) specifically targeting *JMJD2B* (GenePharma, Shanghai, China) comprised the following sequences: *JMJD2B*: siRNA-1, 5′-GCGCAGAAUCUACCAACUU-3′, siRNA-2, 5′-CAAAUACGUGGCCUACAUA-3′; these siRNAs were combined for siRNA transfection. SiRNAs were transfected into HCT116 and SW480 cells using Lipo2000 transfection reagent (Life Technologies, Invitrogen, Carlsbad, CA, USA) in six-well plates with cells at 30% confluence, according to the manufacturer's instructions. The negative control was a nonspecific siRNA (NC-siRNA) (GenePharma, Shanghai, China). Further treatments were applied 24 h after transfection with the siRNAs.

### Cell survival assays, clonogenic assay, and apoptosis detection

For cell survival assays, a cell counting kit-8 (CCK-8; Dojindo, Kumamoto, Japan) was used to examine cell numbers spectrophotometrically, according to the manufacturer's instructions. For the clonogenic assays, at 48 h after transfection with the *JMJD2B* siRNA, cells were trypsinized, counted, and seeded in six-well plates at 600 cells per well. Then at 24 h after seeding, the culture medium was replaced with glucose and amino acids-free media supplemented with or without certain amino acids (Asn, His, and Phe) for 12 h. The media was then replaced with complete growth media for an additional 6 days of cell growth. Colonies were incubated with 4% paraformaldehyde for 10 min. Then, 0.5% crystal violet was used to stain the colonies, and the megascopic colonies were counted. Apoptosis was examined using flow cytometry. *JMJD2B* siRNA-transfected HCT116 and SW480 cells were cultured under the specified conditions and assessed using the Annexin V fluorescein isothiocyanate (FITC)/propidium iodide (PI) double stain assay (BD Pharmingen, San Diego, CA, USA) according to the manufacturer's instructions. All analyses were repeated at least three times.

### Western blotting

Cell extracts were collected and lysed using Radioimmunoprecipitation assay (RIPA) lysis buffer together with a protease inhibitor cocktail (Kangcheng, Shanghai, China). Proteins were separated using SDS-PAGE and then immunoblotted. The primary antibodies used in the present study were: anti-JMJD2B (catalog no. #A301-477A, 1:2000, Bethyl Laboratories, Montgomery, TX, USA); anti-phenylalanine hydroxylase (PAH) (catalog no. #sc-271258, 1:2000, Santa Cruz Biotechnology, Santa Cruz, CA, USA); anti-asparagine synthetase (glutamine-hydrolyzing) (ASNS) (catalog no. #14681-1-AP, 1:2000, Proteintech, Chicago, IL, USA); anti-histidine ammonia-lyase (HAL) (catalog no. # H00003034-M04, 1:2000, Abnova, Taipei, Taiwan); anti-α-tubulin (catalog no. #ab18251, 1:2000, Abcam, Cambridge, UK); anti-LC3B (catalog no. #2775, 1:1000), anti-cleaved Caspase 3 (catalog no. #9664, 1:1000), anti-cleaved Caspase 8 (catalog no. #9496, 1:1000), anti-cleaved Caspase 9 (catalog no. #9505, 1:1000), and anti-cleaved poly (ADP-ribose) polymerase (PARP) (catalog no. #5625, 1:1000) (all from Cell Signaling Technology (Danvers, MA, USA)). Secondary antibodies were conjugated with horseradish peroxidase (HRP) (Kangchen) and the signal was detected using an ECL Kit (SuperSignal West Femto Maximum Sensitivity Substrate, Thermo Scientific, Rockford, IL, USA).

### Quantitative real-time PCR

A reverse transcription reagent kit (Takara, Dalian, China) was used to convert total cellular RNA into cDNA. SYBR Premix® Ex Taq II (Takara) was used to amplify the cDNA on an Applied Biosystems 7900 quantitative PCR system (Applied Biosystems, Foster City, CA, USA). The relative levels of RNA were compared using the 2^-ΔΔCt^ method with *ACTB* (β-actin) as the internal reference gene. The primers used were as follows: for human *JMJD2B*, 5′-TCACCAGCCACATCTACCAG-3′ (forward) and 5′-GATGTCCCCACGCTTCAC-3′ (reverse); for human *LC3B*, 5′- GGCTTTCAGAGAGACCCTGA-3′ (forward) and 5′-GTTTTCTCACACAGCCCGTT-3′ (reverse); for human *ACTB*, 5′-AGAGCCTCGCCTTTGCCGATCC-3′ (forward) and 5′-CTGGGCCTCGTCGCCCACATA-3′ (reverse).

### Chromatin immunoprecipitation (ChIP) assays

ChIP was performed using standard techniques following the manufacturer's protocols (Millipore, Billerica, MA, USA) as described previously 8. Briefly, cells were fixed using 1% formaldehyde, and then lysed and sonicated. The cell lysates were centrifuged, and the supernatant was used for IP. Samples were then incubated with protein A agarose beads and salmon sperm DNA. Thereafter, the anti-JMJD2B antibody (catalog no. #A301-477A, 1:2000, Bethyl Laboratories) and anti-H3K9me3 antibody (catalog no. #ab8898, Abcam) were applied to immunoprecipitate the crosslinked chromatin. We then reversed the crosslinking using a series of washes and extracted the DNA. The DNA was purified for PCR. The primers for the human *LC3B* promoter in ChIP were: 5′-ATCGCATGGTGGTTTACGCACT-3′ (forward) and 5′-AGCCACTAAACTCGCTGGACAA-3′ (reverse).

### Immunohistochemistry (IHC)

Tissues from nude mouse xenograft models of CRC were subjected to IHC to detect LC3B and JMJD2B. JMJD2B and LC3B expression were detected in human CRC tissues. The sections were incubated with the antibodies against JMJD2B (1:200; Bethyl Laboratories) and LC3B (1:200; CST), probed with a secondary antibody, and subsequently mounted with Diaminobenzidine (DAB). The pathological evaluation was conducted in a blinded manner.

### Metabolite-level measurements

Untargeted metabolomic profiles were used to investigate the distribution of metabolites in HCT116 cells after *JMJD2B* knockdown in glucose-containing and glucose-free medium, respectively. Cells were gathered and flash-frozen. The metabolomic analysis was performed by Metabolon (Durham, NC, USA) using their previously described methods 13. For the targeted metabolomics analysis, we used liquid chromatography tandem mass spectrometry (LC-MS/MS) techniques. We prepared a standard curve for each amino acid standard and used the curve to determine the amino acid concentration in each unknown sample.

### Electron Microscopy

We prepared slides for electron microscopy as previously described 14. We used 2.5% glutaraldehyde with 0.1 mol/L sodium cacodylate, and then 1% osmium tetroxide to fix the cells. The samples were hydrated and then embedded. Thereafter, samples were cut into 50 nm sections and stained. Images were obtained using a JEM-1200 electron microscope (JEOL, Tokyo, Japan).

### Statistical analyses

Statistical analyses were performed using the statistical package for social sciences (SPSS) software (IBM Corp., Armonk, NY, USA). Data are shown as the means ± standard deviation (SD). Comparisons of data between two groups were performed using an independent sample t test or an analysis of variance (ANOVA) test, as appropriate. Gene set enrichment analysis (GSEA) was assessed using gsea-3.0 jar.jnlp (The Broad Institute of MIT and Harvard). The correlation between the expression of JMJD2B and LC3B was evaluated using the χ^2^ test. A *p* value of less than 0.05 was accepted as statistically significant.

## Results

### Effect of JMJD2B on amino acid metabolism in CRC Cells

**Figure 1:**
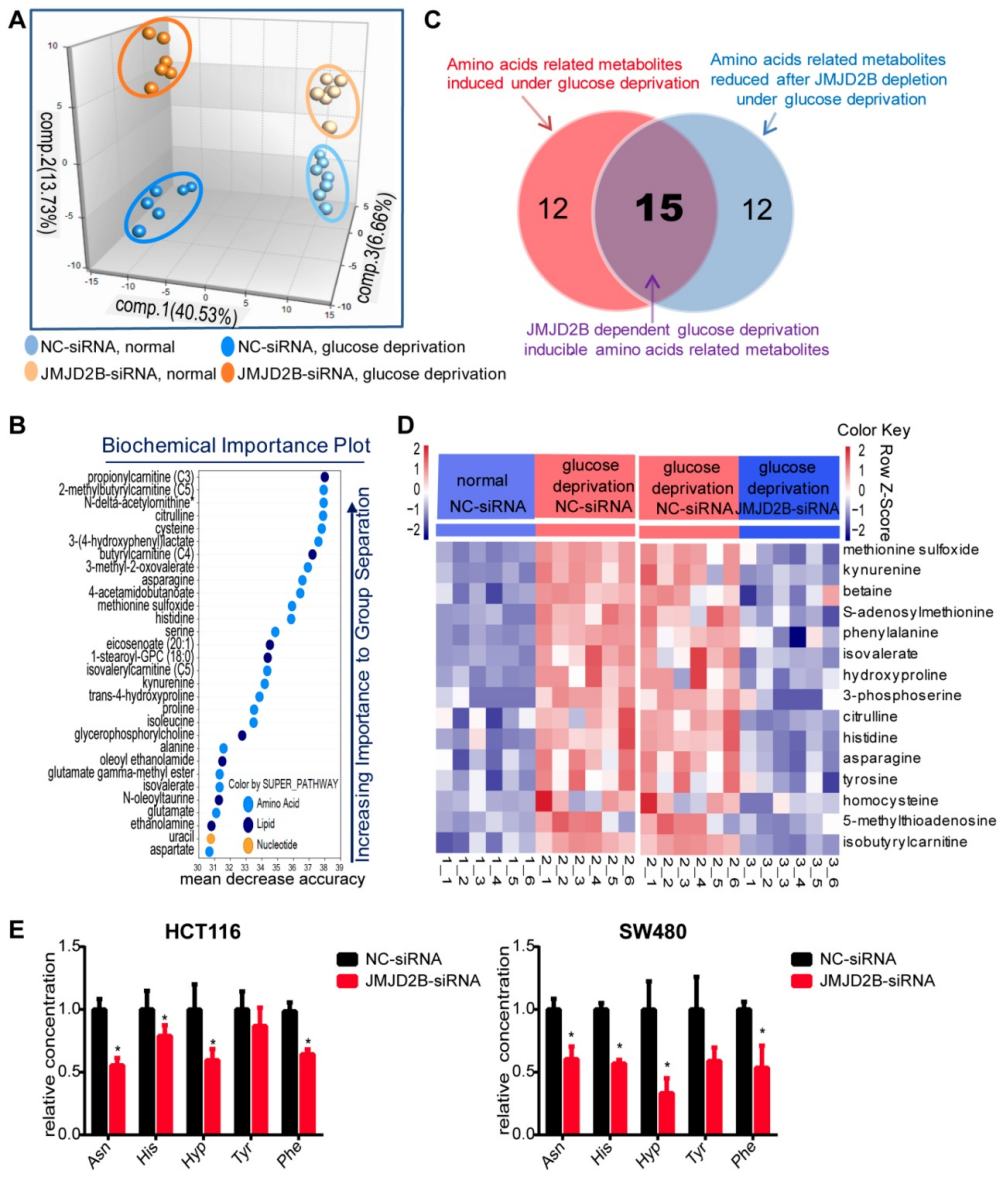
Effect of JMJD2B on amino acid metabolism in CRC Cells. A. Principal component analysis (PCA) of metabolites after *JMJD2B* deletion in HCT116 cells in media with or without glucose. B. Random Forest analysis (RFA) showing a related list of metabolites sorted in order of their significance to differentiate the four groups in HCT116 cells (negative control (NC)-siRNAs or *JMJD2B*-siRNAs in media with or without glucose). C. Venn diagram showing that 27 amino acid-related metabolites were upregulated under glucose deprivation (left red circle), and 27 metabolites were downregulated after *JMJD2B* knockdown (right blue circle) under glucose deprivation conditions. Among these metabolites, 15 were dependent on JMJD2B for glucose-deprivation induction (overlapping region). D. Heatmap of 15 JMJD2B-dependent glucose-deprivation-inducible metabolites (overlapping regions in Figure 1 C). E. Targeted metabolomics analysis for five amino acids (Asn, His, Hyp, Phe, and Tyr) in HCT116 (left) and SW480 (right) cells with *JMJD2B* knockdown. Results are shown as the mean ± SD of four independent samples (*P < 0.05).

Increasing evidence shows that metabolic reprogramming is an important adaptive mechanism for the rapid proliferation of cancer cells in the nutrient-deficiency tumor microenvironment, in which post-translational modifications play a vital role 15-17. Our previous study found that JMJD2B could promote CRC cell survival and regulate multiple cellular processes, especially cellular metabolism processes, under hypoxia or glucose deficiency conditions 8. Thus, we used metabolomic analysis to further explore the effect of JMJD2B on metabolism under glucose deprivation. We observed distinct metabolic reprogramming after *JMJD2B* knockdown *in vitro*, which was more obvious under glucose deprivation conditions, as shown by principal component analysis (PCA) of metabolites (Figure 1A). In addition, random Forest analysis (RFA) indicated that *JMJD2B* suppression led to significant alterations in the levels of amino acids and their metabolites (Figure 1B). Further analysis showed that 27 amino acid-related metabolites were upregulated under glucose deprivation (Figure 1C, left circle), and 27 metabolites were downregulated after *JMJD2B* knockdown (Figure 1C, right circle) under glucose deprivation conditions. Among these metabolites, 15 were dependent on JMJD2B for glucose-deprivation induction (Figure 1C, overlapping region, and shown in the heat-map in Figure 1D), which contained five amino acids (Asn, His, Hyp, Phe, and Tyr). The targeted metabolomics analysis for these five amino acids confirmed that the levels of four of them (Asn, His, Hyp, and Phe) decreased when *JMJD2B* was downregulated (Figure 1E). Overall, these results suggest that JMJD2B has a marked impact on cellular metabolism, especially amino acid metabolism.

### The effects of JMJD2B-dependent glucose deprivation-inducible amino acids on the viability of CRC cells

Considering that promotion of tumorigenesis by JMJD2B is dependent on its impact on amino acid alterations, we tested whether the four amino acids mentioned above have an effect on cancer cell proliferation and survival under glucose deficiency. We found that HCT116 and SW480 cells grown in medium lacking glucose and each amino acid, exhibited significantly reduced proliferation under conditions of *JMJD2B* depletion. Meanwhile, supplementation in the medium with Asn, His, or Phe partly rescued cell proliferation, while Hyp supplementation did not (Figure S1).

**Figure 2:**
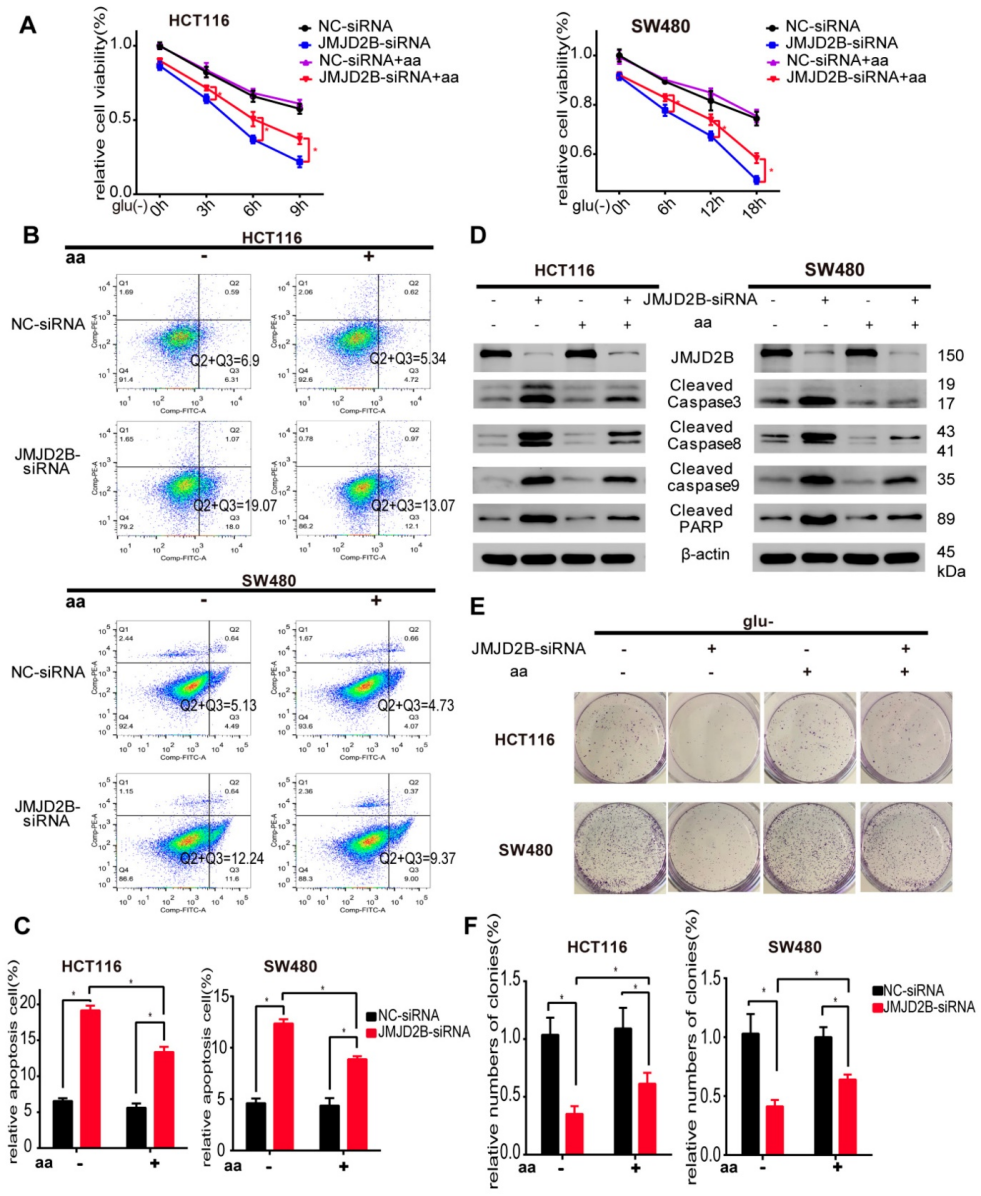
JMJD2B promotes CRC cell survival partly via its effect on amino acids. A. Cell viability analysis of HCT116 and SW480 cells with *JMJD2B* knockdown in the presence or absence of three JMJD2B-dependent glucose deprivation-inducible amino acids (Asn, His, and Phe). Data are presented as the mean percentage ± SD from five independent samples (*P < 0.05). B.C. Apoptosis evaluated using flow cytometry showing the increased apoptosis of HCT116 and SW480 cells after transfection with *JMJD2B* siRNAs in glucose deprivation media. The increased apoptosis was abolished by Asn, His, and Phe treatment. Data from B were quantified in C. The results are shown as the mean ± SD of three independent experiments (*P < 0.05). D. Western blotting analysis of cleaved caspase 3, caspase 8, caspase 9, and PARP in *JMJD2B*-silenced HCT116 and SW480 cells, with or without Asn, His, and Phe treatment. E.F Clonogenic growth experiment of HCT116 and SW480 cells transfected with *JMJD2B* siRNAs or NC siRNAs, with or without Asn, His, and Phe treatment. Quantitative data are presented in the histogram F as the mean ± SD of three independent experiments (*P < 0.05).

We further observed the role of all three amino acids on the regulation of CRC cell survival. As shown in Figure 2A, the *JMJD2B* knockdown‑induced reduction in cell proliferation was partly abrogated by the addition of combined Asn, His, and Phe. In addition, flow cytometry analysis showed that downregulation of JMJD2B-induced apoptosis was abolished by Asn, His and Phe treatment in HCT116 and SW480 cells under the indicated conditions (Figure 2B-C). We then used western blotting to validate that the upregulation of apoptosis indicators after knockdown of *JMJD2B*, including cleaved caspase 3, cleaved caspase 8, cleaved caspase 9, and cleaved PARP, was blocked by supplementation with these three amino acids (Asn, His and Phe) (Figure 2D). Colony formation assays showed that the inhibitory effect of *JMJD2B* knockdown was partly reversed using Asn, His, and Phe supplements (Figure 2E-F). Thus, supplementation with Asn, His, and Phe, partly restored cell survival after *JMJD2B* knockdown under glucose and amino acid deprivation, which indicated that JMJD2B promotes the survival of CRC cells under glucose deprivation by maintaining the intracellular level of amino acids (Asn, His, and Phe).

### *JMJD2B* silencing inhibits CRC cell autophagy via epigenetic downregulation of *LC3B*

Amino acid uptake and biosynthesis, as well as the degradation of proteins, contribute to the maintenance of cellular amino acid levels 9; however, the mechanism by which JMJD2B maintains the levels of amino acids (Asn, His, and Phe) in CRC cells under glucose deprivation is unknown. We therefore tested the Asn, His, and Phe uptake in *JMJD2B*-silenced cells under glucose deprivation, and revealed that *JMJD2B* silencing did not affect Asn and Phe uptake, and only enhanced His uptake slightly in HCT116 cells (Figure S2A), which should increase intracellular His level when *JMJD2B* is knocked down, which is inconsistent with the metabolomics analysis in Figure 1E. These results suggested that alterations to amino acid uptake may not be the main mechanism by which JMJD2B sustains intracellular Asn, His, and Phe levels.

To gain insights into the biosynthesis pathway, GSEA was applied. Enrichment plots of GSEA showed no correlation between JMJD2B and the Asn, His, or Phe metabolism gene signatures in our previous gene microarrays with* JMJD2B* silencing 3 (Figure S3A) and in TCGA datasets (GSE39582 and GSE17536, Figure S3B-C). We further observed the mRNA levels of key genes in Asn metabolism (*ASNS*, encoding asparagine synthetase (glutamine-hydrolyzing)), His metabolism (*HAL*, encoding histidine ammonia-lyase), and Phe metabolism (*PAH*, encoding phenylalanine hydroxylase) using qPCR 18-20, which showed that *JMJD2B* silencing did not affect *HAL, ASNS*, and *PAH* expression both in HCT116 and SW480 (Figure S2B). We also detected the protein levels of those three genes; no significant change in *ASNS* and *PAH* expression was observed with JMJD2B silencing (Figure S2C), and we did not detect the HAL protein band using western blotting, possibly because of its low expression in HCT116 and SW480 (data not shown). These data indicated that JMJD2B-induced maintenance of Asn, His, and Phe levels may not act via regulation of their biosynthesis.

**Figure 3:**
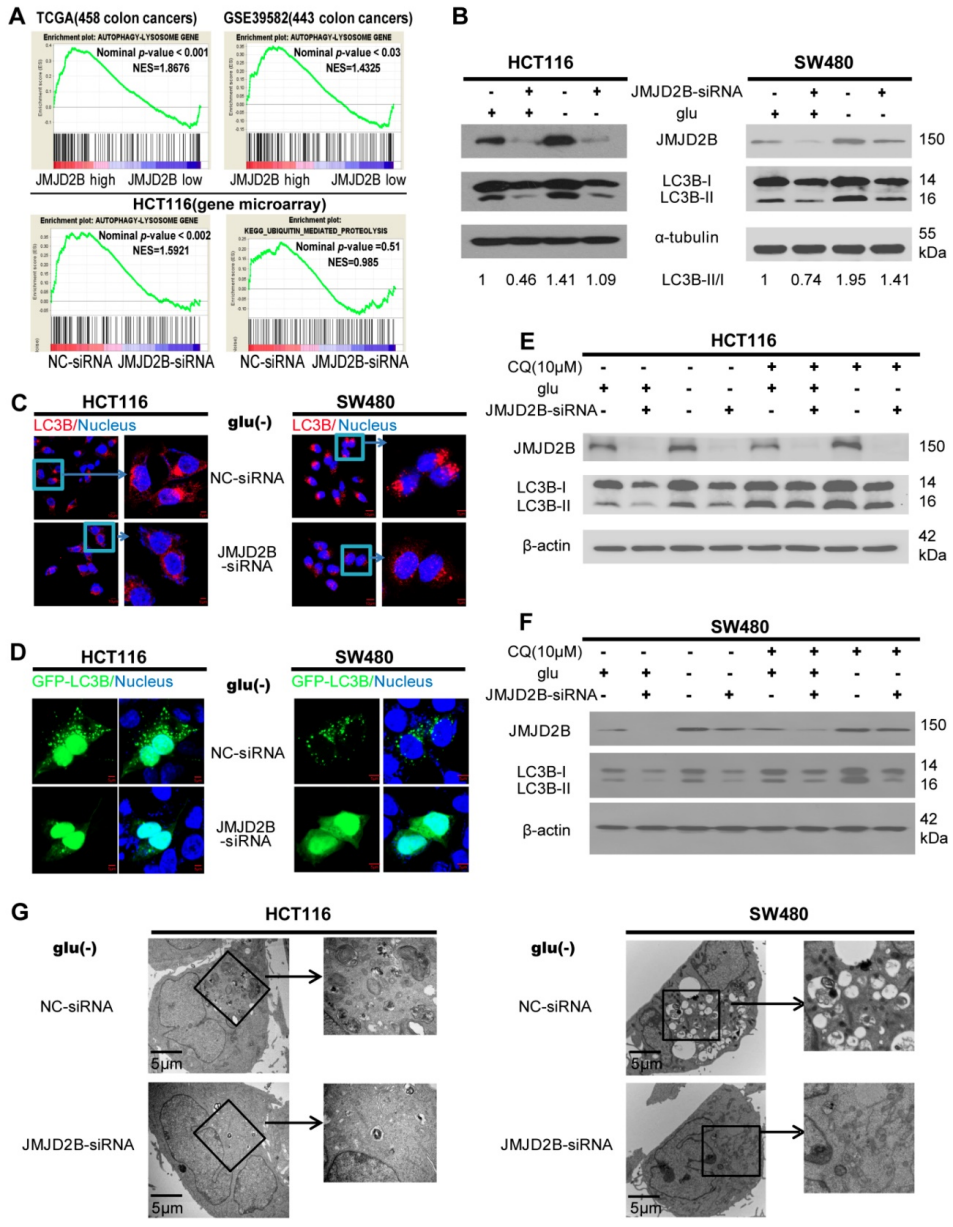
*JMJD2B*-silencing inhibits CRC cell autophagy. A. The positive association between JMJD2B and autophagy-related gene signatures was demonstrated using GSEA analysis in the TCGA (459 colon cancer) and GSE39582 (443 colon cancer) data sets (upper panel). The correlation between JMJD2B and autophagy-related gene signatures or the ubiquitin-proteasome system was conducted using GSEA analysis in gene microarrays with *JMJD2B* silencing (lower panel). B. Western blot of LC3B levels under normal growth conditions and glucose deprivation conditions in HCT116 and SW480 cells with downregulation of *JMJD2B* expression. C. Immunofluorescence staining of LC3B in HCT116 and SW480 cells transfected with NC-siRNA or *JMJD2B*-siRNA after glucose deprivation. Scale bar: 5 μm. D. Immunofluorescence staining of GFP-LC3B in HCT116 and SW480 cells after blockage of *JMJD2B* after glucose deprivation. E.F. Western blot of LC3B levels in HCT116 and SW480 cells co-cultured with CQ and transfected with NC-siRNA or *JMJD2B*-siRNA, with or without glucose. G. Transmission electron microscopy (TEM) analysis of HCT116 and SW480 cells silenced for *JMJD2B* expression after glucose deprivation.

Protein degradation has been suggested to be important for amino acid homeostasis 21-24, including macroautophagy (hereafter referred to as autophagy) and the ubiquitin-proteasome system. GSEA was conducted to investigate the relationship between JMJD2B and autophagy-related gene signatures in two independent colon cancer datasets (TCGA (458 colon cancer cases) and GSE39582 (443 colon cancer cases)) (Figure 3A), and a positive relation between JMJD2B and autophagy-related gene signatures was demonstrated, similar our previous gene microarrays from *JMJD2B*-silenced cells (Figure 3A) 3. However, no correlation was found between JMJD2B and the ubiquitin-proteasome system (Figure 3A and Figure S2D). These results led us to hypothesize that the degradation of proteins via autophagy might contribute to JMJD2B-induced maintenance of intracellular Asn, His, and Phe levels. To examine the role of JMJD2B in autophagy, we performed autophagy functional assays in CRC cells with *JMJD2B* silencing. Decreased conversion of LC3B-I to LC3B-II was detected in the *JMJD2B*-silenced HCT116 and SW480 cells (Figure 3B). Immunofluorescence in *JMJD2B*-silenced HCT116 and SW480 cells indicated decreased endogenous LC3B staining under glucose deprivation (Figure 3C). Next, we detected the formation of GFP-tagged LC3B-positive autophagosomes, and found that suppression of *JMJD2B* expression decreased the GFP-LC3B punctate cells under glucose deprivation in HCT116 and SW480 cells (Figure 3D). Furthermore, we performed autophagic flux analysis using chloroquine (CQ), an inhibitor of autophagosome-lysosomal fusion, and found that CQ blocked autophagic flux in HCT116 and SW480 cells transfected with *JMJD2B*-siRNA under glucose deprivation (Figure 3E-F). In addition, transmission electron microscopy showed a decrease in the formation of autophagic vesicles in the *JMJD2B*-siRNA-transfected HCT116 and SW480 cells (Figure 3G). Taken together, our data supported the hypothesis that *JMJD2B* silencing inhibited autophagy in CRC cells.

**Figure 4:**
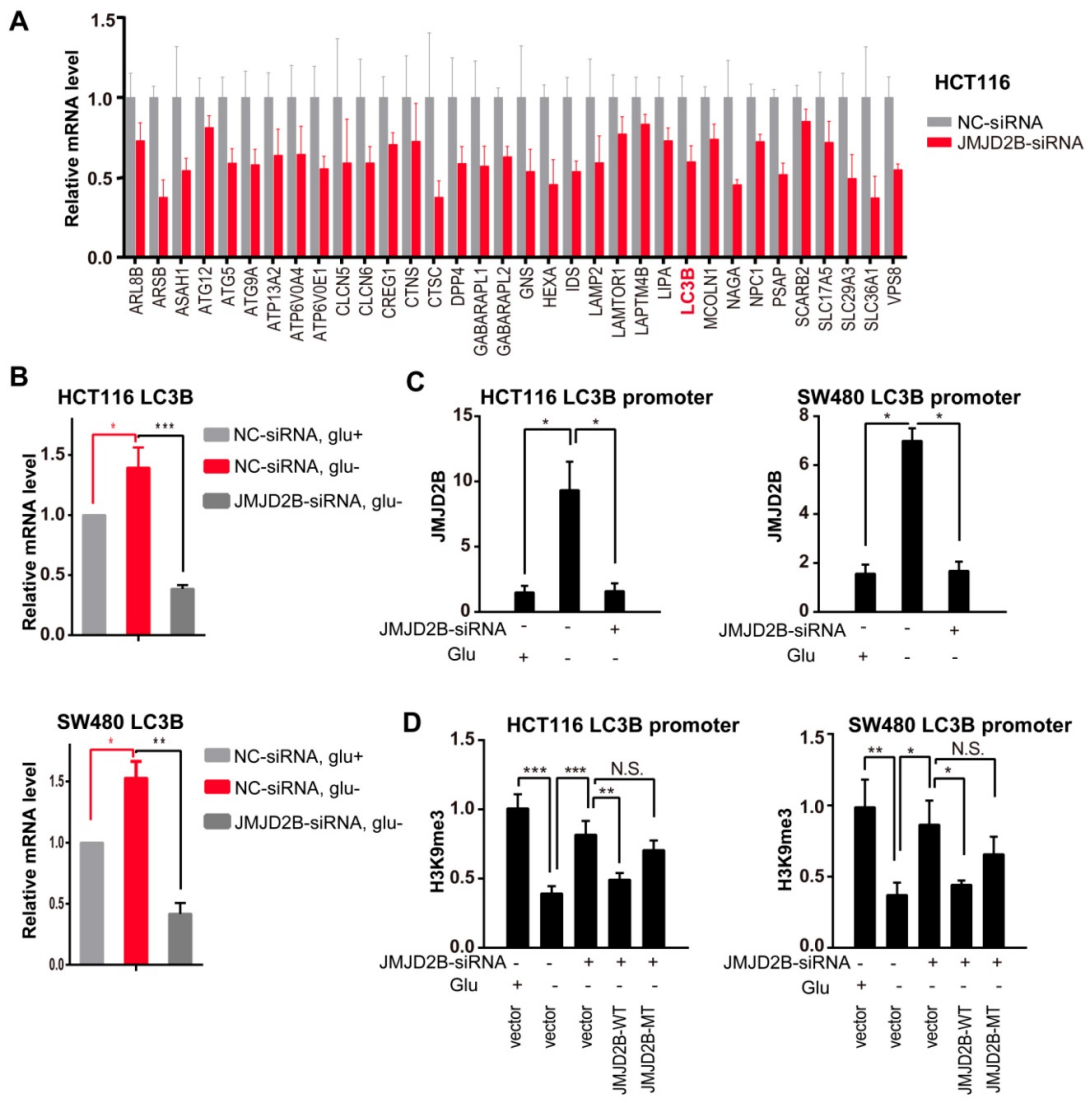
Transcriptional upregulation of *LC3B* by JMJD2B during glucose deprivation. A. *JMJD2B* knockdown in HCT116 cells caused coordinate downregulation of autophagy lysosome genes. Values were taken from an Affymetrix gene microarray in triplicate experiments; *P* < 0.05 for each gene. B. Real-time PCR validation of the mRNA levels of *LC3B* in HCT116 and SW480 cells. Data are normalized to the expression level of *ACTB* (*P < 0.05, **P < 0.01, ***P < 0.001). C. Quantitative ChIP PCR analysis showing JMJD2B binding to the* LC3B* promoter in HCT116 and SW480 cells with *JMJD2B* knockdown and in control cells (*P < 0.05). D. Quantitative ChIP PCR analysis of the levels of H3K9 tri-methylation binding to the* LC3B* promoter in HCT116 and SW480 cells. Data are displayed as the fold change calculated relative to the controls (*P < 0.05, **P < 0.01, ***P < 0.001).

We next explored the underlying molecular mechanism. Recently, histone post-translational modifications, including dimethylated H3K9 (H3K9me2), have been recognized as key regulators of autophagy 25. When autophagy is induced, G9A (the H3K9 methyltransferase) leaves the promoters of *LC3B*, which allows demethylation of H3K9, thereby activating *LC3B* expression 26, which is evolutionarily conserved throughout eukaryotes and plays a key role in autophagosome formation. However, the histone demethylase responsible for this step during autophagy has not been identified. JMJD2B, a histone demethylase, is responsible for demethylation of H3K9 me2/3; therefore, we tried to determine whether JMJD2B functioned in the epigenetic regulation of autophagy. In line with this, knockdown of *JMJD2B* in HCT116 cells resulted in prominent repression of the autophagy pathway (gene microarray, Figure 4A), including* LC3B.* Then, we validated the mRNA expression of *LC3B* using qRT-PCR. As shown in Figure 4B, the *LC3B* mRNA level decreased substantially after knockdown of *JMJD2B.* In addition, western blotting analysis also showed that *JMJD2B* knockdown could decrease both the LC3B-I and LC3B-II levels (Figure 3B).

Furthermore, we used a ChIP assay to detect the association of JMJD2B and H3K9me3 with the *LC3B* promoter. The results showed an increased binding of JMJD2B to the *LC3B* promoter and a reduction in H3K9me3 levels on the *LC3B* promoter under glucose deficiency, while these changes were attenuated after *JMJD2B* knockdown (Figure 4C-D, Figure S4A-B). To test whether the decrease in H3K9me3 intensity was catalyzed by JMJD2B directly, the siRNA-resistant JMJD2B wild-type plasmid (JMJD2B-WT) and the H189A/E191Q mutant plasmid (JMJD2B-MT), a catalytically inactive mutant without lysine de-methylation activity 27, 28, were transfected into JMJD2B-knockdown cells and cultured under glucose deprivation conditions. In contrast to JMJD2B WT, a nonsignificant change in recruitment was detected in the JMJD2B MT group in JMJD2B-knockdown cells under glucose deficiency (Figure 4D, Figure S4B). These data indicated that JMJD2B-mediated regulation of *LC3B* is dependent on its demethylase activity.

### LC3B is involved in JMJD2B mediated-amino acid alterations and promotion of tumorigenesis* in vitro*

**Figure 5:**
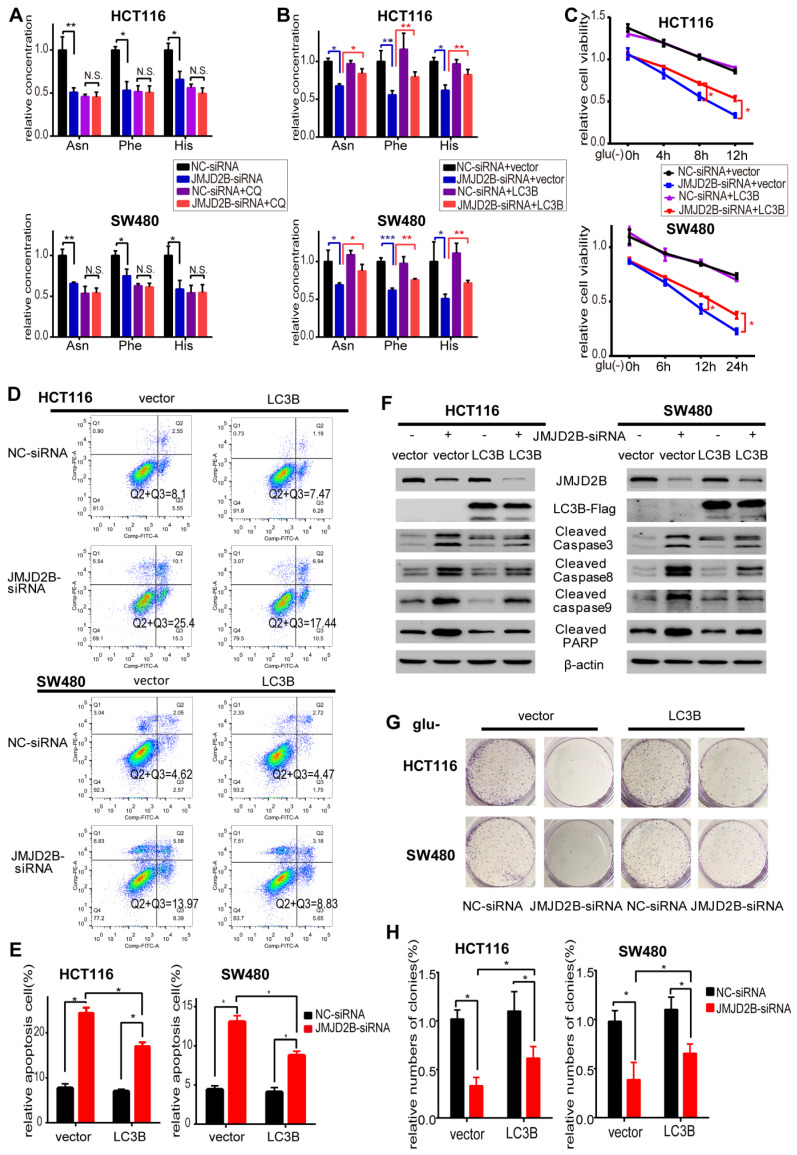
JMJD2B-regulated autophagy via *LC3B* is essential for sustaining intracellular Asn, His, Phe levels and promoting CRC cell survival. A. *JMJD2B* knockdown caused a decrease in Asn, His, and Phe levels in HCT116 and SW480 cells, while the inhibitory effects of JMJD2B-silencing were not observed in cells treated with the autophagy inhibitor CQ (*P < 0.05, **P < 0.01). B. *JMJD2B* knockdown caused a decrease in Asn, His, and Phe levels in HCT116 and SW480 cells, while these effects were rescued by overexpression of *LC3B* (*P < 0.05, **P < 0.01, ***P < 0.001). C. Cell viability analysis of HCT116 and SW480 cells showed that overexpressing of *LC3B* reversed *JMJD2B* silencing-induced decreases in cell viability. Data are presented as the mean percentage ± SD from five independent samples (*P < 0.05). D.E. Apoptosis evaluated using flow cytometry showing that overexpressing of *LC3B* decreased CRC cell apoptosis in response to *JMJD2B* silencing. Quantitative data are presented in a histogram (E). The results are displayed as the mean ± SD of three independent experiments (*P < 0.05). F. Immunoblotting for cleaved caspase 3, caspase 8, caspase 9, and PARP levels in *JMJD2B*-silenced HCT116 and SW480 cells treated with or without *LC3B* overexpression. G.H. Clonogenic growth experiments of HCT116 and SW480 cells showing the promoting effects of *LC3B* overexpression on the *JMJD2B* silencing-induced reduction in colony-forming capacity. Quantitative data are presented in a histogram (H, mean plus SD of three independent experiments (*P < 0.05)).

We next hypothesized that JMJD2B-mediated amino acid alterations might act via autophagy. To test this hypothesis, we assessed the intracellular Asn, His, and Phe levels in the presence of the autophagy inhibitor CQ under glucose deprivation conditions, and observed that *JMJD2B* knockdown decreased the levels of these three amino acid in HCT116 and SW480 cells under glucose deprivation; however, the inhibitory effects of *JMJD2B*-silencing were not observed in cells treated with CQ (Figure 5A).

These data supported the view that autophagy is involved in JMJD2B's effects on intracellular Asn, His, and Phe levels. JMJD2B activated autophagy via epigenetic regulation of *LC3B*; therefore, we next examined whether LC3B participated in JMJD2B-mediated alteration of amino acids and promotion of tumorigenesis, using an LC3B overexpression plasmid transfected into JMJD2B-knockdown CRC cells. As shown in Figure 5B, the decreases in Asn, His, and Phe levels caused by JMJD2B-siRNAs in HCT116 and SW480 cells were rescued by transfecting cells with the *LC3B* plasmid*.* In addition, the inhibitory effects of *JMJD2B*-silencing on cell viability and colony-forming capacity were attenuated by LC3B plasmid transfection (Figure 5C, 5G-H). In addition, LC3B plasmid transfection decreased the proportion of apoptotic cells and the levels of apoptosis indicators in response to *JMJD2B* knockdown (Figure 5D-F). These data supported the view that JMJD2B-mediated alterations of amino acids and promotion of tumorigenesis function partly through regulation of LC3B.

### The function of LC3B in JMJD2B-mediated tumorigenesis *in vivo*

**Figure 6:**
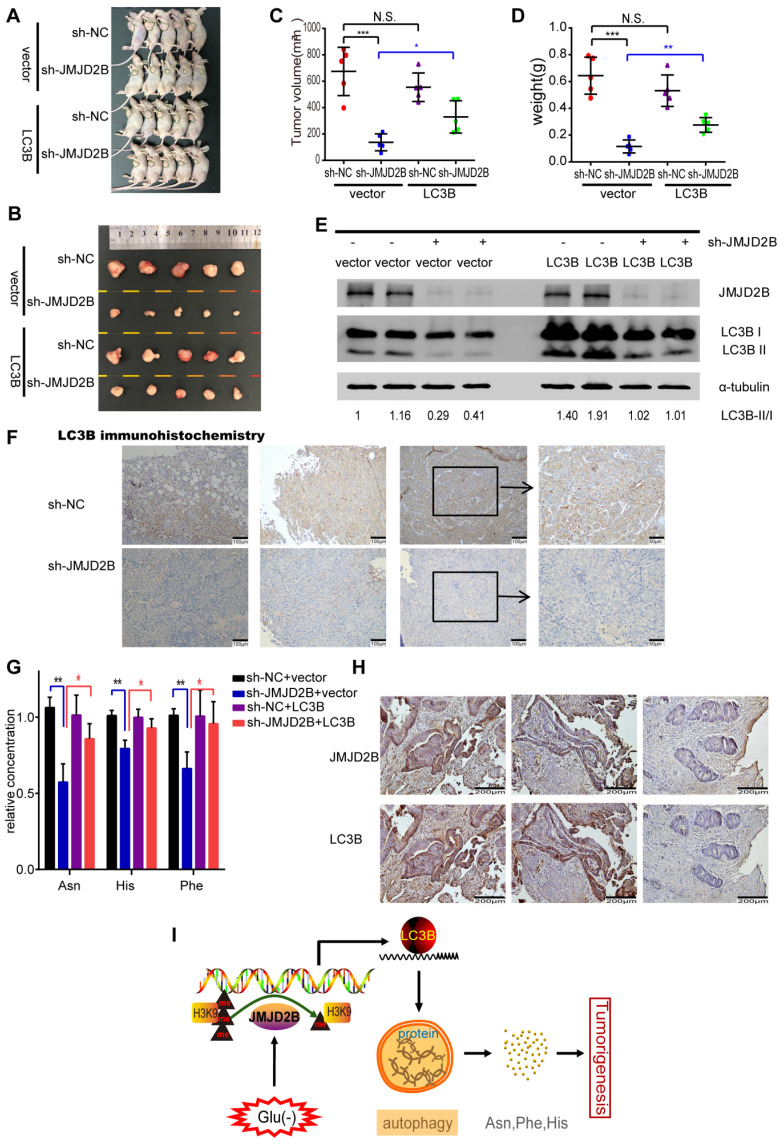
The function of LC3B in the JMJD2B-mediated tumorigenesis in vivo. A-D Tumors from different groups were dissected and photographed (A, B), and the tumor volumes and weights were measured (C, D). Data are presented as the mean ± SD. (*P < 0.05, **P < 0.01, ***P < 0.001). E.F. JMJD2B and LC3B protein levels in the xenografts were detected using western blotting (E) and immunohistochemistry (F). G. Levels of amino acids in tumor lysates derived from xenografts were measured. Data are presented as the mean ± SD. (*P < 0.05, **P < 0.01). H. Representative immunohistochemistry of JMJD2B (upper) and LC3B (lower) proteins in CRC tissues. I. Schematic model showing that JMJD2B activates autophagy via epigenetic regulation of *LC3B*, resulting in the maintenance of intracellular Asn, His, and Phe levels, which consequently promote the survival of CRC cells upon glucose deprivation.

To address the oncogenic role of LC3B in JMJD2B-mediated tumorigenesis *in vivo,* we further generated a CRC xenograft model and tested the levels of amino acids and autophagy in tumor tissues in nude mice bearing HCT116 cells in different experimental conditions. Our previous study showed the inhibiting effect of *JMJD2B* silencing on CRC xenografts growth* in vivo* 4. In line with that research, the tumor volume and tumor weight in the *JMJD2*B shRNA group were significantly decreased compared with those of the control shRNA group (*P* < 0.05) (Figure 6 A-D), and these decreases were blocked in the group stably overexpressing LC3B established using a lentivirus targeting the *LC3B* gene (Figure 6A-D). Furthermore, we detected the LC3B protein levels in tumor lysates derived from xenografts. As shown in Figure 6 E, similar results were observed to the *in vitro* results: The transition from LC3B I to LC3B II and the total amount of LC3B were reduced upon knockdown o*f JMJD2B*. Using immunohistochemistry, we also observed that the LC3B level was diminished in the xenograft tumor tissues upon *JMJD2B* knockdown (Figure 6F). We further tested the levels of amino acids in the xenograft tissues, and observed that knockdown of *JMJD2B* resulted in significantly decreased of levels of Asn, His, and Phe, which was rescued in the group stably overexpressing LC3B (Figure 6G). The data was consistent with the* in vitro* results.

### Correlation of JMJD2B expression and LC3B expression in human CRC tissues

To determine the association between JMJD2B and LC3B expression in human CRC tissues, immunohistochemistry of JMJD2B and LC3B was performed in 60 CRC specimens. High-expression of JMJD2B and LC3B proteins were detected in 35 (58.3%) and 31 (51.7%) of the 60 CRC specimens, respectively. The results showed that the samples with high-expression of JMJD2B displayed a high level of LC3B, and the samples with JMJD2B lower expression exhibited weakly staining for LC3B (Figure 6H). The data was statistically significant (*P* < 0.001), which further confirm the correlation between JMJD2B and LC3B.

Thus, we proposed that JMJD2B activates autophagy via epigenetic regulation of LC3B, resulting in the maintenance of intracellular Asn, His, and Phe levels, which consequently promote the survival of CRC cells upon glucose deprivation (Figure 6I).

## Discussion

In the present study, profound metabolic reprogramming was detected during *JMJD2B* knockdown under glucose deficiency in CRC cells, particularly those involving amino acid metabolites. Furthermore, we showed that *JMJD2B* knockdown resulted in decreased intracellular amino acid levels (Asn, Phe and His), which partly depended on the regulation of autophagy under glucose-deprivation. JMJD2B could enhance CRC cell survival under glucose starvation through its regulation of amino acid levels (Asn, Phe and His). In addition, *JMJD2B* knockdown inhibited CRC cell autophagy by epigenetically downregulating *LC3B* expression, with a consequent negative impact on the survival of CRC cells upon glucose deprivation.

JMJD2B plays an oncogenic role in different cancers, such as gastric cancer, lung cancer, renal cancer, bladder cancer, and myeloma cells 29-32. Our previous study indicated that JMJD2B is overexpressed in CRC tissues, correlates positively with a deeper depth of invasion and advanced clinical stages, and promotes CRC development 3. The oncogenic potential of JMJD2B in CRC was also observed by Liu et al., who showed that high expression of JMJD2B correlated positively with the lymph node status, Dukes' classification, and tumor invasion of patients with CRC 33. We have reported that JMJD2B regulates multiple cellular processes under the metabolic stress, especially cellular metabolism process under hypoxia 3, 4. Thus, we speculated that JMJD2B might be involved in the metabolic changes of tumor cells in CRC. Using metabolic profiling, the effects of JMJD2B on metabolites of CRC cells were analyzed. The metabolites were significantly different after inhibiting the expression of *JMJD2B* in CRC cells, especially amino acids and their related metabolites. Further analysis showed that the glucose-deprivation-induced increases in amino acids (Phe, His, Asn and Hyp) were abrogated upon *JMJD2B* silencing.

Insufficient angiogenesis means that cancer cells in solid tumors are usually under hypoglycemic and other cellular metabolic stresses, which result in cell death. Therefore, cancer cells undergo a series of compensatory mechanisms, including post-translational modifications of histones and metabolic reprogramming, to overcome metabolic stress-induced cell death 34, 35. Recently, many non-glutamine amino acids have been discovered to have a critical role in the adaptation of tumor cells to metabolic stress or chemotherapy 9, 12, 18, 36. Certain amino acid biosynthesis pathways are activated in tumor cells to promote the production of special amino acids (such as serine, aspartic acid, Asn, and glycine) 9, 37. Indeed, decreasing the bioavailability of Asn could significantly reduce the invasive ability of tumor cells by affecting the synthesis of epithelial-mesenchyme transition (EMT)-related proteins 18. Intracellular Asn can promote the proliferation of tumor cells, and maintain their amino acid homeostasis and metabolism 12. Phe and Tyr deficiency or His deficiency could diminish the proliferation of cancer cells and induce tumor cell apoptosis 38-46. To clarify whether the changes in intracellular amino acid levels regulated by JMJD2B affect the survival of CRC cells, we detected the role of four amino acids (Phe, His, Asn, and Hyp) in cell viability. Consistent with previous studies, we found that supplementation with Asn, His, and Phe, but not Hyp, partly restored cell survival after *JMJD2B* knockdown. This indicated that JMJD2B sustained the cellular level of amino acids in CRC cells upon glucose deficiency, thus driving the malignancy of CRC.

It has been reported that intracellular protein degradation pathways occupy an important position among the factors that affect the level of amino acids in tumor cells. Protein degradation could provide amino acids for oxidative and biosynthetic reactions during nutritional deficiency 21-24, 47, 48. Ubiquitination proteasomes and autophagy are the two major protein degradation systems 49. Inhibition of ubiquitin proteasomes or autophagy could decrease the cellular levels of amino acids 22, 23, 50. In addition,* in vivo* experiments showed that the levels of amino acids in plasma could be regulated by autophagy related 5 (ATG5) deficiency 47 or by inducing liver autophagy 51. Similarly, glucose deficiency can induce autophagy, increase the proportion of amino acids entering the tricarboxylic acid cycle, and provide energy for tumor cells, which may be the reason for the increase in the levels of certain amino acids induced by glucose deficiency 52-54. Using GSEA, we found that JMJD2B was positively associated with autophagy-related genes, but not with ubiquitin proteasomes in CRC; therefore, we hypothesized that JMJD2B might regulate autophagy to maintain amino acids levels. The findings of the present study showed that inhibition of JMJD2B under glucose deficiency could inhibit autophagy in CRC cells. We also found that the inhibitory effects of JMJD2B-silencing on the intracellular Asn, His, and Phe levels were not observed in cells treated with CQ (Figure 5 A), which supported the view that autophagy is involved in the JMJD2B's effects on the levels of these three amino acids.

Many studies have indicated that post-translational modifications of histones are involved in the regulation of autophagy, such as H3K4me3, H4K20me3, H3K9me2, H4K16ac, and H3K56ac 25. G9A (methylating H3K9), MOF (acetylating H4K16), and SIRT1 (deacetylating H4K16) are involved in the regulation of autophagy 25, 55. JMJD2B, which demethylates H3K9me3/2, might participate in the regulation of autophagy. Demethylation of H3K9 occurs when autophagy is induced, thereby activating *LC3B* expression 26. However, the histone demethylase responsible for this process during autophagy has not yet been reported. We further explored the mechanism by which JMJD2B could regulate *LC3B*, and observed that JMJD2B could bind to the *LC3B* promoter to de-methylate H3K9me3. Using a plasmid carrying a catalytically inactive mutant without lysine de-methylation activity, we demonstrated that the decrease in intensity of H3K9me3 binding to the promoter of *LC3B* was catalyzed by JMJD2B directly. A recent study revealed that suppression of JMJD2B could inhibit autophagy through regulation of *ULK1* and *ATG16L1*, and showed increase killing in Nutlin-treated A549 cells, one of the non-small-cell lung cancer cell lines 56. These results are consistent with the effect of JMJD2B on autophagy in our study. Next, we revealed that transfection of *LC3B* only partly rescued the inhibitory effects of knockdown of *JMJD2B* on the levels of intracellular Asn, His, and Phe, and the cell survival (Figure 6C-D and 6G), suggesting that JMJD2B might affect the expression of other autophagy genes to regulate autophagy and amino acid levels, which is consistant with the results in Figure 4A. Our results suggested that LC3B participates in JMJD2B-mediated alteration of amino acids and promotion of tumorigenesis. Further research to underscore the physiological relevance and the effects on other autophagy genes is needed.

In conclusion, amino acid alterations caused by JMJD2B might be important for the survival of CRC cells adapting to glucose deficiency. In response to glucose deprivation, JMJD2B promotes autophagy to produce three intracellular amino acids (Asn, His and Phe), which could promote the survival of CRC cells through epigenetic regulation of *LC3B*. Our results may provide a better understanding of the molecular mechanism leading to CRC, and identify JMJD2B as a potential target for CRC treatment. Future work to determine the therapeutic or diagnostic value of JMJD2B, as well as to identify the factors that result in JMJD2B recruitment to the promoters of genes, is necessary.

## Supplementary Material

Supplementary figures.

## Abbreviations

CRC: colorectal cancer
Asn: asparagine
Phe: phenylalanine
His: histidine
Hyp: hydroxyl-proline
KDM4B: lysine-specific demethylase 4B
TCA: tricarboxylic acid cycle
CCK-8: cell counting kit-8
LC-MS/MS: liquid chromatography tandem mass spectrometry
GSEA: Gene set enrichment analysis
SD: standard deviation
PCA: principal component analysis
RFA: Random Forest analysis
TCGA: The Cancer Genome Atlas
CQ: chloroquine
LC3B: microtubule associated protein 1 light chain 3 beta
