# Supplementary material for: JMJD2B-induced amino acid alterations enhance the survival of colorectal cancer cells under glucose-deprivation via autophagy
Source: Theranostics. 2020 Apr 27;10(13):5763–77. doi: 10.7150/thno.38087 (PMC7254993; doi:10.7150/thno.38087)
Supplement: Supplementary file 1 — Supplementary figures. [file thnov10p5763s1.pdf]

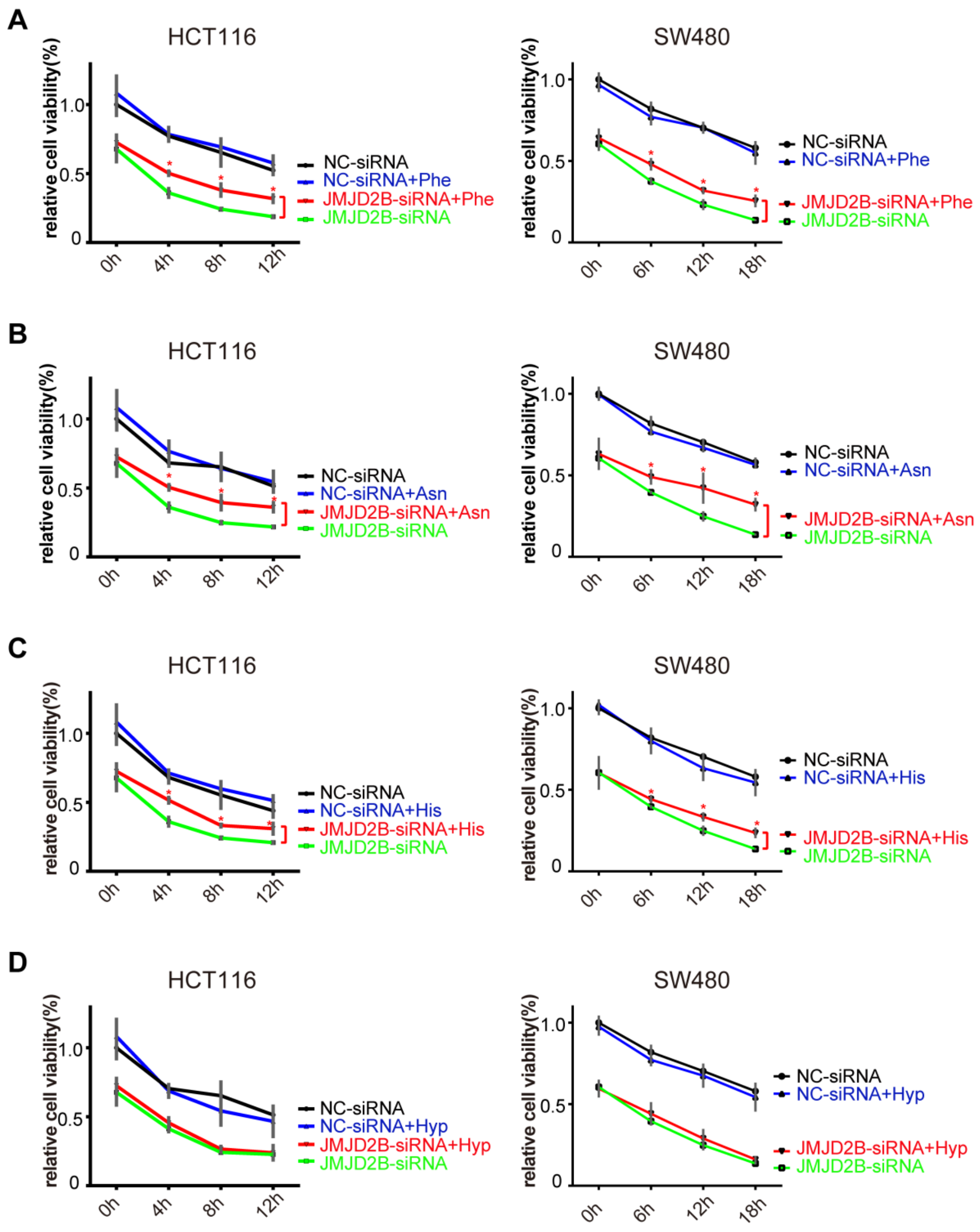

Figure S1 The effects of Phe, Asn, His, and Hyp on CRC cell viability

A–D Viability analysis of HCT116 (left) and SW480 cells (right) transfected with *JMJD2B* siRNAs or NC siRNAs in the presence or absence of Phe (A), Asn (B), His (C), and Hyp (D) under glucose and amino acid deprivation conditions. Data are presented as mean percentage ± SD from five independent samples (*$P < 0.05$).

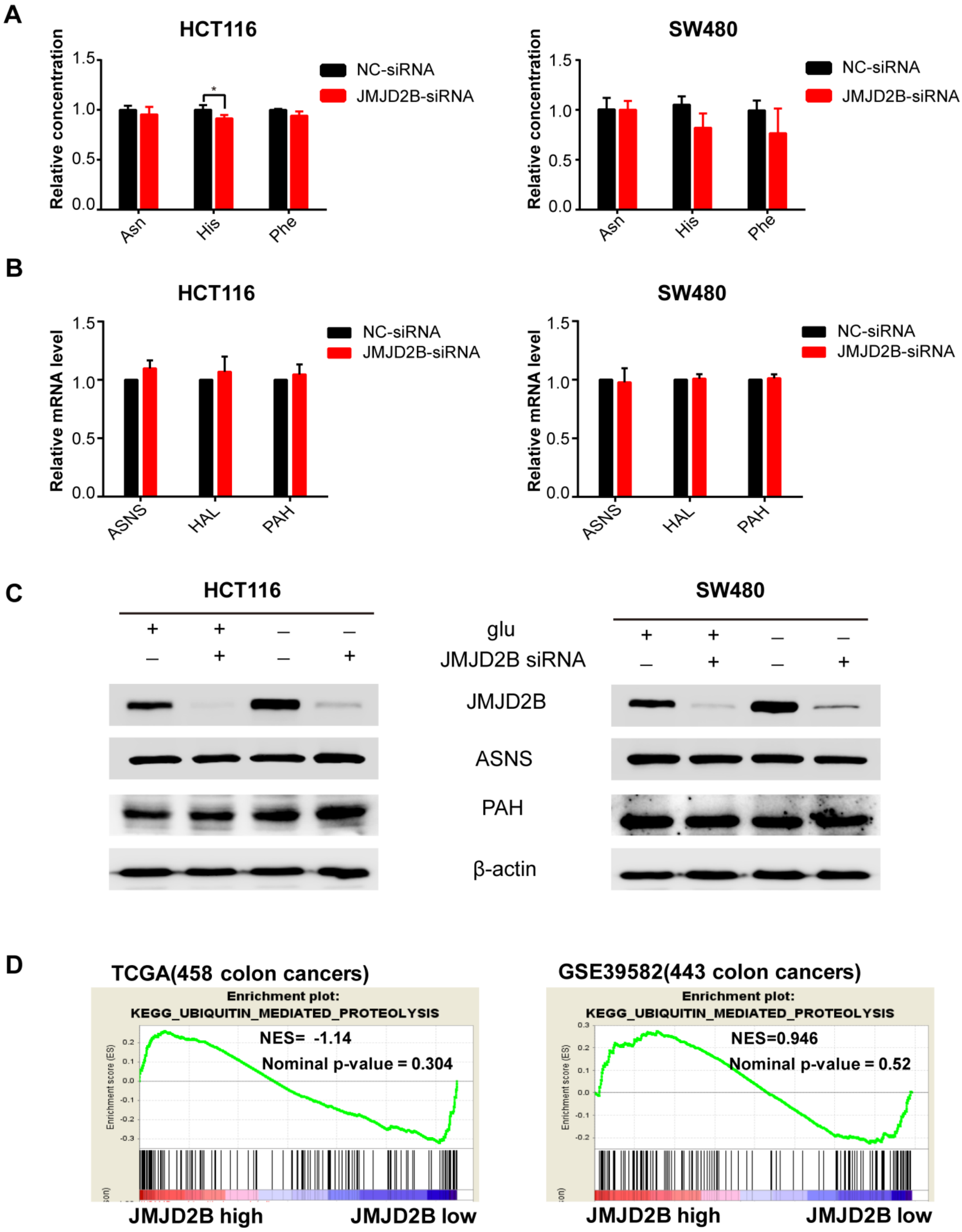

Figure S2 JMJD2B sustains Asn, His, and Phe levels in CRC cells by a mechanism unrelated amino acids uptake, biosynthesis and ubiquitin-mediated proteolysis.

A. Asn, His, and Phe uptake was measured as fold change in extracellular amino acids in HCT116

and SW480 cells following transfection with *JMJD2B* siRNAs or NC siRNAs. Media was changed 1 h before media samples were harvested for analysis.

B. Real-Time PCR analysis of the expression levels of *ASNS*, *HAL*, and *PAH* in HCT116 and SW480 cells. Data were normalized to the level of *ACTB*, and presented as fold changes relative to the mRNA levels in NC-siRNA-treated cells.

C. Western blot of the levels of *ASNS* and *PAH* in HCT116 and SW480 cells.

D. GSEA analysis of the relationship between JMJD2B and Ubiquitin-mediated proteolysis gene signature.

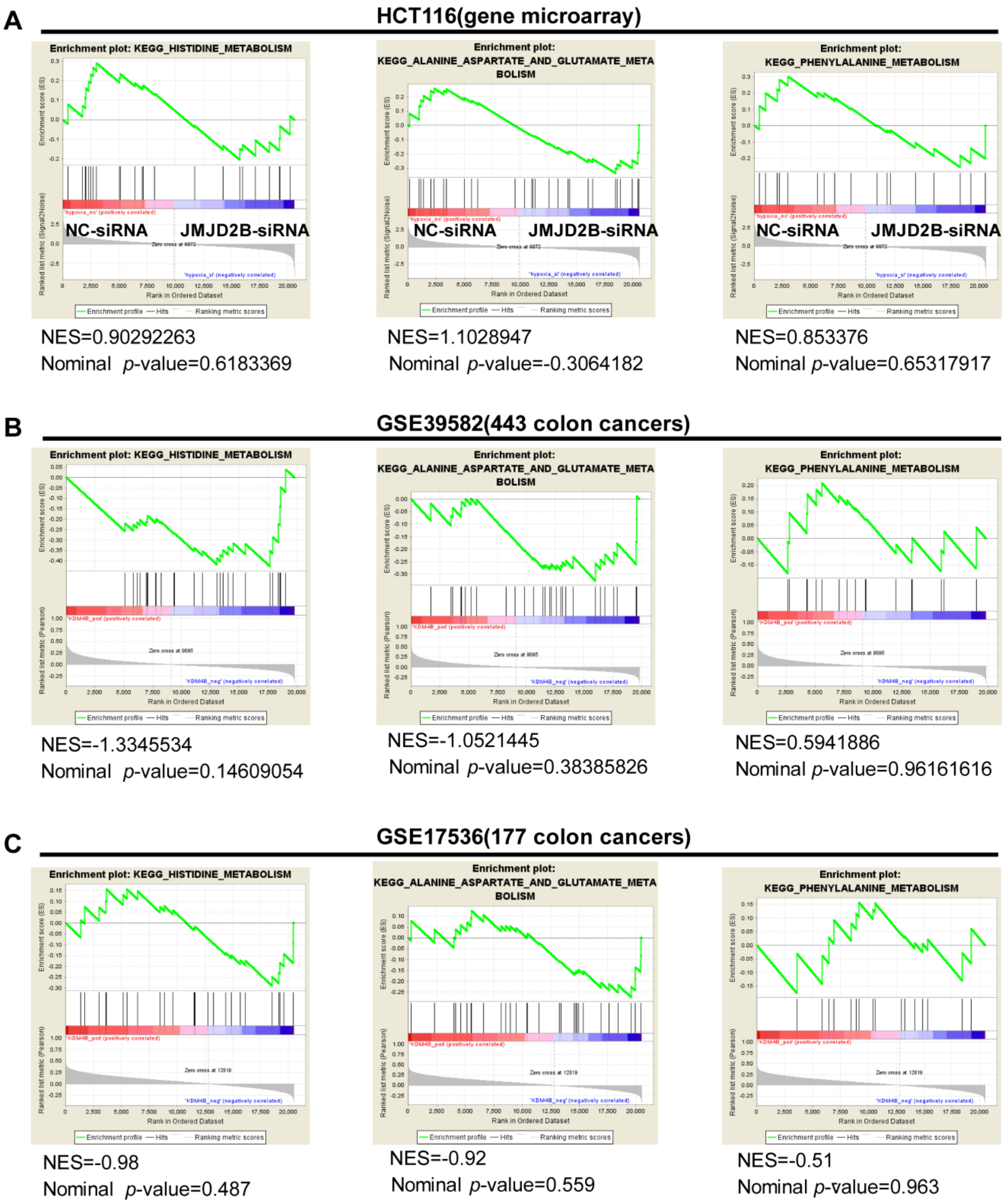

Figure S3 GSEA analysis of the relationship between JMJD2B and Asn, His, and Phe metabolism gene signatures

The relationship between JMJD2B and Asn, His, and Phe metabolism gene signatures was analyzed using GSEA analysis in gene microarrays with *JMJD2B* silencing (A), GSE39582 (B. 443 colon cancers) and GSE17536 (C. 177 colon cancers).

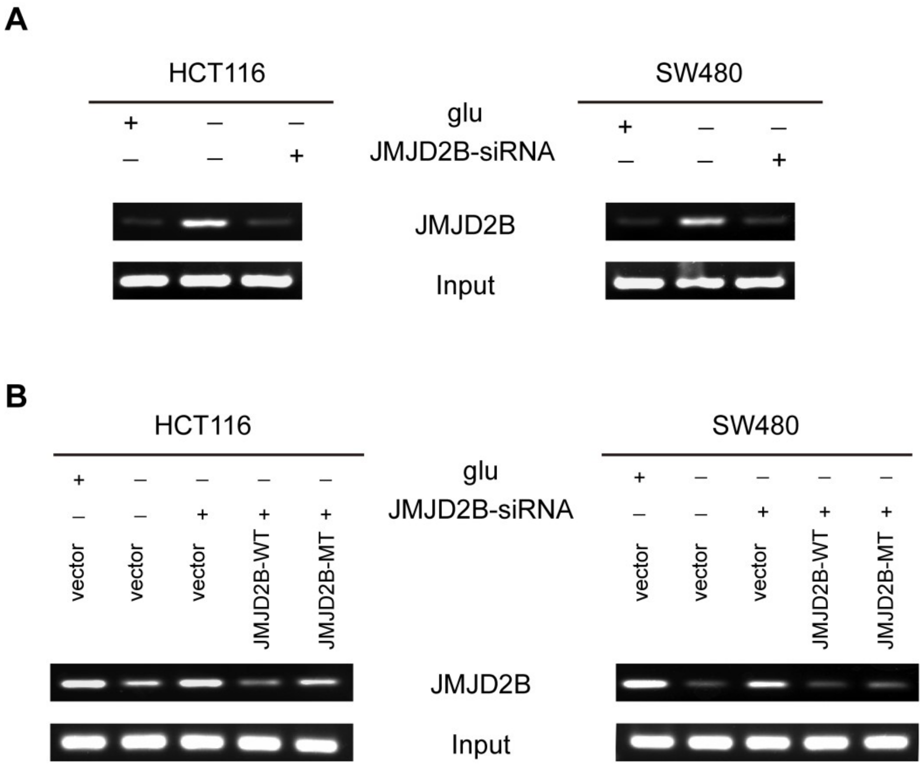

Figure S4 Agarose gel analysis of the ChIP-PCR products

A. Agarose gel analysis of the ChIP-PCR products showing JMJD2B binding to the *LC3B* promoter in HCT116 and SW480 cells in different groups.

~~D~~. Agarose gel analysis of the ChIP-PCR products of the levels of H3K9 tri-methylation binding to the *LC3B* promoter in HCT116 and SW480 cells in different groups.
